# Efficacy of a digital cognitive behavioral therapy for insomnia in people with low back pain: a feasibility randomized co-twin and singleton-controlled trial

**DOI:** 10.1186/s40814-022-01087-z

**Published:** 2022-06-14

**Authors:** Kevin K. N. Ho, Milena Simic, Marina B. Pinheiro, Christopher B. Miller, Manuela L. Ferreira, Ronald R. Grunstein, John L. Hopper, Juan R. Ordoñana, Paulo H. Ferreira

**Affiliations:** 1 Faculty of Medicine and Health, The University of Sydney, Susan Wakil Health Building, Sydney, NSW 2050 Australia; 2 Institute for Musculoskeletal Health, The University of Sydney and Sydney Local Health District, Sydney, Australia; 3 Big Health Inc., San Francisco, USA; 4 Big Health Inc., London, UK; 5 Sleep and Circadian Neuroscience Institute, Nuffield Department of Clinical Neurosciences, University of Oxford, Oxford, UK; 6 Institute of Bone and Joint Research, The Kolling Institute, Northern Clinical School, Faculty of Medicine and Health, The University of Sydney, Sydney, Australia; 7 CIRUS, Centre for Sleep and Chronobiology, Woolcock Institute of Medical Research, University of Sydney and Royal Prince Alfred Hospital, Sydney, Australia; 8 Centre for Epidemiology and Biostatistics, Melbourne School of Population and Global Health, The University of Melbourne, Melbourne, Australia; 9 Murcia Twin Registry, Department of Human Anatomy and Psychobiology, University of Murcia, and IMIB-Arrixaca, Murcia, Spain

**Keywords:** Sleep, Low back pain, Randomized control trial, Twins, Digital, Insomnia

## Abstract

**Background:**

Digital cognitive behavioral therapy for insomnia (CBT-i) in people with low back pain (LBP) may be efficacious in improving both sleep and pain; and twin trial designs provide greater precision of treatment effects by accounting for genetic and early environmental factors. We aimed to determine the feasibility of a trial investigating the efficacy of a digital CBT-i program in people with comorbid symptoms of insomnia and LBP, in twins and people from the general community (singletons).

**Methods:**

Thirty-two twins (16 pairs) and 66 singletons with comorbid symptoms of insomnia and LBP (> 6 weeks duration) were randomized to digital CBT-i (intervention) or educational program (control) for 6 weeks. The digital CBT-I, Sleepio (developed by Big Health Inc.), was an online interactive, automated, personalized course comprising of six sessions, once a week. The education program was six emails with general sleep information, once a week. Participants were blinded to their group allocation and offered the alternative intervention at the completion of the study. Feasibility outcomes included recruitment and follow-up rates, data collection and outcome measure completion, contamination (communication about trial interventions), acceptability (adherence), credibility, and participants’ experience of the intervention.

**Results:**

Sixteen out of 722 contacted twin pairs were recruited (recruitment rate = 2.2%). Twins were recruited between September 2015 and August 2018 (35 months) and singletons between October 2017 and Aug 2018 (10 months). Follow-up rates for post-intervention and 3-month follow-up were 81% and 72% for twins and 82% and 78% for singletons respectively. Adherence rates (percentage of sessions completed out of six) for the digital CBT-i were 63% for twins and 55% for singletons. Contamination (speaking about the study to each other) was present in two twin pairs (13%). Written or verbal feedback (*n* = 21) regarding the digital CBT-i intervention from participants were positive (*n* = 11), neutral (*n* = 5), or negative (*n* = 6).

**Conclusions:**

Online CBT-i was received favorably with people with comorbid symptoms of insomnia and LBP. While the online data collection was successful, strategies need to be implemented to improve adherence, follow-up, control group credibility (for digital CBT-i), and twin recruitment rates (for twin trials).

**Trial registration:**

Australian New Zealand Clinical Trials Registry (ACTRN12615000672550). Registered 29 June 2015

**Supplementary Information:**

The online version contains supplementary material available at 10.1186/s40814-022-01087-z.

## Key messages regarding feasibility

- The feasibility of digital CBT-i for people with LBP and the recruitment of twins in an RCT are uncertain.
- For digital CBT-i, feasibility goals were met for data collection, but not for follow-up and adherence. For an RCT involving twins, feasibility goals for contamination of intervention were met but not for recruitment.
- This study identified strategies to improve adherence, follow-up, control group credibility for digital CBT-I for people with LBP, and recruitment rates for twins for the main study and future similar studies.

## Background

Low back pain (LBP) is the lead cause of years lived with disability in Australia and worldwide [1], and its impact on disability-adjusted life years is expected to continually increase with the aging population [2]. Recent studies on LBP have encouraged the need for clinicians and researchers to assess and address modifiable comorbidities [3], including insomnia. People with insomnia have twice the odds of reporting chronic LBP (OR = 1.99, 95% CI [1.79–2.21] [4], and the presence of comorbid insomnia in people with LBP is associated with higher pain intensity (mean difference = 13.0/100, 95% C I [1.5–24.5]) [5], and outpatient costs [6]. Because the relationship between insomnia and pain intensity has been regarded as bidirectional [7–9], the management of insomnia in people with LBP has the potential to improve both sleep and pain.

International guidelines recommend cognitive behavioral therapy (CBT-i) as the first line of care for insomnia [10–15]. A recent systematic review conducted by our group concluded that the use of face to face CBT-i for insomnia for people with comorbid chronic LBP reduced insomnia severity (Pittsburgh Sleep Quality Index = − 3.90/21, 95% CI [− 5.65, − 2.15]) and pain intensity (visual analog scale = − 8.49/100, 95% CI [− 16.46, − 0.53] ) [16]. However, patient access to face to face CBT-i can be problematic [17] due to its high cost and limited availability of trained therapists [18, 19], which need to be addressed for people with limited access to specialize healthcare facilities [20].

Digital CBT-i has proven successful in delivering insomnia treatment by increasing accessibility and lowering costs [18, 21]. Sleepio, developed by Big Health Inc., is an online application which has improved insomnia symptoms (effect size = 1.1–1.5 vs control) in randomized control trials [21, 22]. However, the acceptability (adherence) to digital CBT-i may differ in people with comorbid LBP and insomnia (e.g., if people believe that an LBP focused treatment is better to manage both conditions) and impact the efficacy of the intervention to improve insomnia and pain in this population.

There has been growing interest from the musculoskeletal research community for designing randomized co-twin controlled trials [23–25]. This design allows optimal matching to control for genetic and early life environmental factors which may contribute to more precise treatment effects [26]. Genetics may influence people’s responses to treatment, as LBP (21-67%) [27] and insomnia (38-59%) have high heritability rates [28], and familial factors are known to influence people’s response to common treatments for LBP such as physical activity [29]. Within-pair analysis in this trial design may provide up to 14 times the statistical power compared to the general population (singletons) due to optimal matching of age, sex, family background, and genetics [30, 31]. However, feasibility aspects such as recruitment rates of twins from twin registries e.g., Twins Research Australia (TRA), and potential design limitations e.g., “contamination of intervention” (communication between participants about trial interventions) are yet to be evaluated.

The aim of this feasibility randomized co-twin and singleton-controlled trial evaluating the efficacy of a digital cognitive behavioral therapy for insomnia in people with low back pain were to investigate: (1) the rate of recruitment of adult twins and singletons from the general population to participate in the trial (number of people randomized), (2) the feasibility of online data collection and outcome measure completion, (3) contamination of intervention among twins, and (4) acceptability, credibility, and participants’ experience with Sleepio.

## Methods

### Study design

The protocol of this feasibility randomized co-twin and singleton-controlled trial for people with comorbid symptoms of insomnia and chronic LBP (SleepBack) has been previously published [32]. There are two deviations from the protocol, (1) the inclusion of a singleton cohort and (2) broadening of the inclusion criteria for symptoms of insomnia, and they are described below. The present study has been approved by the Research Ethics Committee of the University of Sydney (2015/386) and registered (ACTRN12615000672550). The protocol has been written following the SPIRIT statement [33], and findings reported according to CONSORT [34] statement and the TIDieR checklist [35].

### Participants

A total of 32 twins (16 pairs) and 66 singletons were recruited between November 2015 and August 2019. This was a deviation from the original protocol, where only 30 twins (15 pairs) were proposed to be recruited. There were two reasons for this change: (1) we observed a lower than expected recruitment rate for twins and (2) to allow a comparison of the feasibility of recruiting samples of twins and singletons.

The process of trial recruitment differed for twins and singletons. Twins were recruited in collaboration with TRA, an organization that operates as a twin registry and national twin research center. TRA invited twins through email to participate in the trial [32] as well as in a twin observational study for LBP (AUTBACK study [36]). In consideration of recruitment costs, this was initially a targeted approach of twins who indicated having LBP in a 2014 TRA health survey. At the end of the participant information sheet, twins were invited to answer a preliminary screening questionnaire. This preliminary screening questionnaire confirmed whether interested twins had current LBP and sleep problems. Complete twin pairs (i.e., both twins responding to the invitation) who expressed their interest were contacted by the TRA to confirm their preliminary eligibility and consent.

For singletons, we invited twins from incomplete twin pairs (i.e., individuals who were interested but their twin was not), as well as people from the general community via newsletters (e.g., NSW Seniors Cards’ newsletter), posters, websites, and social media (e.g., Facebook). Twins and singletons who were interested in participating in the trial were contacted by the research team either by phone or email and given the study Participant Information Statement via REDCap (Research Electronic Data Capture) hosted at the University of Study [37]. Those who agreed to participate underwent formal comprehensive self-reported screening via REDCap.

Clarification of screening responses were followed up by telephone and email. To be included in the study as a twin pair, both twins needed to meet all the criteria and had their zygosity ascertained. The inclusion criteria and exclusion criteria were identical to the protocol (Table 1) [32], except for one modification which had been approved after the publication of the protocol. In the initial protocol, a cut-off score of ≤ 16 was used as an indicator of probable insomnia; however, this stricter cut-off (higher scores indicated better sleep) was relaxed as it excluded a significant number of participants. Singletons only needed to individually satisfy the same modified criteria.

**Table 1:** Protocol inclusion criteria, exclusion criteria, and patient measures

**Inclusion**	
1) Aged between 18 and 65 years 2) Current LBP of at least 6 weeks duration and not currently seeking care for LBP 3) At least 3/10 pain on the numerical pain scale 4) Have current access to the internet 5) A score of ≤ 24 on the Sleep Condition Indicator, which is indicative of sub-clinical insomnia symptoms in accordance to the Diagnostic and Statistical Manual of Mental Disorders Fifth Edition	
**Exclusion**	
1) Had known or suspected serious spinal pathology (e.g., fracture, metastatic, inflammatory or infective diseases and widespread neurological disorder) 2) Had spinal surgery within the last 12 months 3) Were using prescribed treatments for insomnia or depression 4) Were pregnant or lactating 5) Presented with severe symptoms of depression (score > 10), anxiety (> 7), or stress (> 12) according to the Depression Anxiety Stress Scales (DASS-21) 6) Reported poor physical or mental health (self-report 5-point Likert scale) 7) Reported substance use disorder 8) Were shift workers	
**Protocol patient measures**	
1) Pain self-efficacy questionnaire (PSEQ) 2) Patient-specific functional scale (PSFS) 3) Numerical pain rating scale (NRS) (Scale 0-100) 4) Roland Morris disability questionnaire (RMDQ) 5) International Physical Activity Questionnaire Short Form (IPAQ-SF) 6) Depression, Anxiety and Stress Scale - 21 Items (DASS-21) 7) Insomnia Severity Index (ISI), 8) Sleep efficiency (SE)	

### Assessments

The patient outcomes have been described in detail in the protocol (Table 1) [32]. Patient outcomes were assessed via online questionnaires at baseline, post-intervention, and 3-month follow-up. The Pittsburgh Sleep Quality Index (PSQI) [38] and Sleep Condition Indicator (SCI) [39] were also collected, and approved in the original ethics application, but were not mentioned in the protocol manuscript.

Participants were asked to complete the Intervention Credibility Scale 1-week post-allocation. All questionnaires were conducted via online self-reported questionnaires through REDCap. Reminders were sent to participants at 7 days by email and phone text message and at 2 weeks by phone call.

At post-intervention, blinding was assessed with the question “Which intervention did you receive” with the response options being “real (experimental) intervention” or “sham (control) intervention.” Twins also answered the questions regarding contamination of intervention at post-intervention and were phone interviewed on their opinion regarding the sleep intervention and their experience with the study.

### Randomization and blinding

Twins were block randomized so that each twin within a pair was allocated to a different intervention group. Singletons were randomized in a 1:1 ratio to ensure both groups had the same number of allocated participants. Randomization was performed by a computer-generated random allocation schedule by a remote researcher. The remote researcher was blinded from the participant characteristics and the allocation was concealed from participants, the main assessor, and the trial statistician of the study. All participants were contacted via phone to commence their interventions and blinded to whether they received the real intervention or sham. Twin participants commenced their interventions in a synchronized manner and were asked not to discuss with their co-twin about the study intervention they were receiving.

### Intervention and control groups

The study groups have been described in detail in our protocol [32]. The experimental group received digital CBT-i in the form of an interactive, automated, personalized course comprising of six sessions, once a week (Sleepio [21, 40]) (Appendix 1). The control group received a general digital education program in the form of six weekly emails to match the experimental intervention period and frequency of online interactions with participants. Each weekly newsletter content was different, with information regarding sleep mainly extracted from the Sleepio library. The sleep education alone is known to not be effective at improving insomnia [41, 42] and hence used as the control. In our Participant Information Statement, participants were informed that they would be offered the alternative intervention at the completion of the study if they wished so.

### Outcomes and criteria for feasibility

#### Recruitment rate

Records were kept for the number of twins approached by the TRA. The number of twins and singletons screened by the researchers, eligible for the trial, and recruited were recorded. The feasibility criteria were that (1) ≥ 10% of twins contacted by the TRA were recruited and (2) ≥70% of eligible twin pairs consented to be included in the trial [32]. No recruitment rate criteria were set for singletons.

#### Data collection and outcome measure completion

The number of missing items for each study questionnaire at baseline and follow-up were used to determine data completion. Questionnaire reminder emails, phone messages( and phone calls were utilized at 7 and 14 days after the assessment was due. Participants who did not submit their questionnaire answers were counted as lost-to-follow-up. The reasons and number of lost-to-follow-up and withdrawals at each phase of the study were also noted. During the end of study phone interview, twin participants were asked about their experience with the online data collection method, including whether they had any difficulties in answering the questionnaires. The feasibility criteria were based on the PEDro scale [43], with ≤ 20% missing data for outcome measures and ≥ 85% follow-up rate [32].

#### Contamination of intervention

While the randomized co-twin control design has many advantages, there is a potential for twins to indirectly or directly inform their co-twin on intervention allocation (contamination of intervention) and compromise the integrity of participant blinding. This may happen despite allocation being concealed to participants as they may share the details of their intervention. All participants were asked to not discuss the nature of their intervention with any other participant (e.g., their twin) for the duration of the study. Possible contamination of intervention was assessed via online questionnaires which asked participants if they discussed with their co-twin about the interventions, they were confident that their intervention was not known by their co-twin, they were aware of the intervention their co-twin received, and if they changed their behavior as a consequence of knowing their twin’s intervention. Contamination of intervention was also evaluated in the phone interviews with the following question “Did you speak with your twin about your intervention or work out what your twin received?”. We also assessed how often twins spoke to each other and whether they lived together. The pre-specified feasibility criterion was ≤ 15% of the twins being aware of the intervention their co-twin was receiving [32]. This criterion was based on the ≥ 85% follow-up rate on the PEDro scale [43], as contaminated twin pairs may be considered a data lost to follow-up.

#### Acceptability, credibility, and participants’ experience of the intervention

For the digital CBT-i group, the following information on the acceptability and experience with the intervention were assessed: percentage of sessions attempted out of six (adherence) and whether they would recommend the intervention to another person (at the 3-month follow-up). For the educational control group, there were difficulties in ascertaining adherence as the email newsletters did not have a tracking mechanism. Intervention credibility was assessed at 1-week post randomization, by using four modified prospective questions from Borkovec and Nau [44] to investigate whether our experimental and control intervention were equally credible. Opinions regarding the intervention were asked during the follow-up questionnaire and phone interview. The feasibility criterion for adherence was ≥ 75% participants completing at least four of the six sessions [32], based on trials conducted for CBT-i (Sleepio) [21].

#### Data analysis

Descriptive statistics were used to detail the baseline characteristics of twins and singletons. Analyses were focused on the variability of the data and assessed by 95% confidence intervals (CI). The results for all feasibility outcomes were detailed separately for twins and singletons for comparison.

### Feasibility results

#### Flow of participants and recruitment rate

In the first round of recruitment (September 2015 to April 2016), 719 potential twin pairs were directly contacted by TRA, and 30 pairs met the preliminary screening questionnaire and had their details forwarded to the researchers. A total of 18 pairs completed the formal comprehensive screening questionnaire, where four pairs met the complete inclusion criteria and were recruited. In the second round of recruitment (August to December 2016), the insomnia inclusion criterion was modified and another eight pairs were recruited. In the final round of recruitment (July to August 2018), participants who had completed the observational AUTBACK study [36] were contacted, and the final four pairs were recruited. Costs were only pertained to the 2015–2016 recruitments which involved TRA directly contacting participants and totaled AUD 5956.50.

**Fig. 1:**
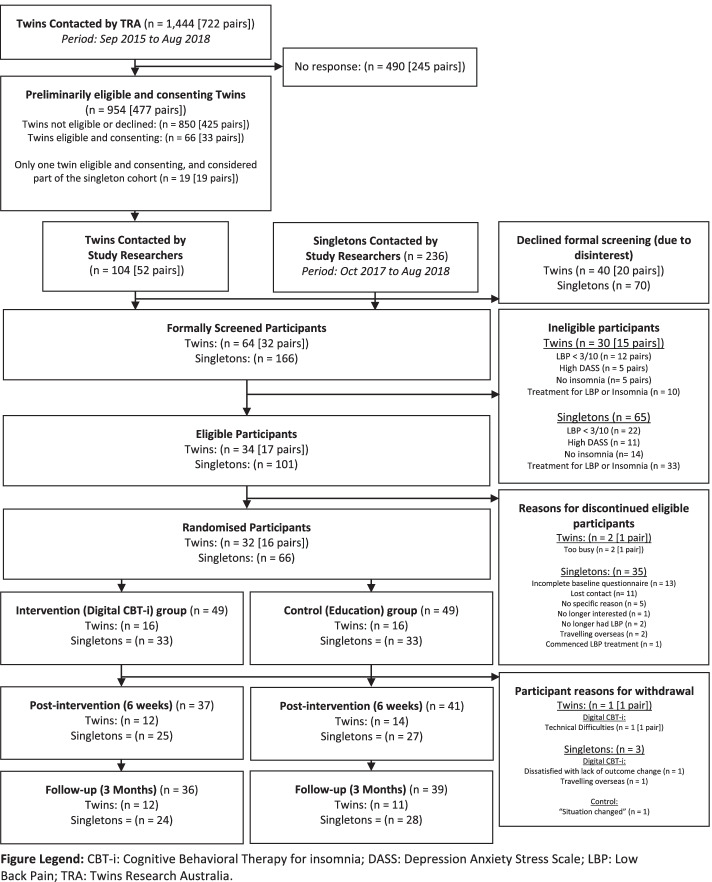
Flowchart of participants

In total, from September 2015 to August 2018, 722 twin pairs were contacted directly by the TRA, 52 pairs expressed interest and were contacted by researchers, and 32 pairs were screened. Of the 17 twin pairs who were eligible after answering the formal comprehensive screening questionnaire, 16 pairs were recruited (94.1%) as one pair stated they were not available to participate in the trial (recruitment rate 2.2%) (Fig. 1). Fifteen of these pairs were monozygotic, and one pair was dizygotic. Therefore, the feasibility criteria of “≥ 10% of twins contacted by the TRA were recruited” was not met, but the criteria of “recruiting ≥70% of eligible twin pairs” was. The characteristics of the participants are described in Table 2.

**Table 2:** Characteristics of participants in the included sample

Characteristic	Total (digital CBT-i) *n* = 49	Total (control) *n* = 49	Twins (digital CBT-i) *n* = 16	Twins (control) *n* = 16	Singletons (digital CBT-i) *n* = 33	Singletons (control) *n* = 33
Age						
Mean (SD)	52.7 (12.5)	53.1 (11.5)	48.9 (10.4)	48.9 (10.4)	54.6 (13.2)	55.2 (11.6)
Range	18–65	22–65	29–64	29–64	18–65	22–65
Sex (female) (%)	33 (67%)	33 (67%)	13 (81%)	13 (81%)	20 (61%)	20 (61%)
Low back pain measures, mean (SD)						
Pain self-efficacy (PSEQ)^a^	45.2 (10.5)	38.8 (11.0)	48.5 (7.6)	43.8 (9.4)	43.5 (11.5)	36.3 (11.0)
Disability (RMDQ)^b^	6.6 (4.2)	8.3 (4.9)	5.1 (2.4)	4.6 (3.2)	7.3 (4.8)	10.1 (4.6)
Pain (NPS)^c^	47.6 (20.6)	51.1 (18.8)	45.3 (20.8)	48.5 (19.3)	48.8 (20.7)	52.3 (18.7)
Function (PSFS)^d^	4.7 (1.9)	4.9 (1.7)	5.5 (2.1)	5.4 (1.8)	4.2 (1.6)	4.6 (1.7)
Sleep measures						
Insomnia severity (ISI)^e^	13.1 (5.1)	12.6 (4.5)	11.4 (4.4)	11.3 (3.4)	13.9 (5.3)	13.3 (4.8)
Sleep condition (SCI)^f^	12.5 (4.1)	12.5 (4.9)	13.5 (3.8)	14.0 (2.7)	12.3 (4.2)	12.1 (5.3)
Sleep quality (PSQI)^g^	9.6 (3.3)	9.6 (3.2)	9.1 (3.2)	9.6 (3.1)	9.8 (3.4)	9.6 (3.2)
Depression Anxiety Stress Scale						
Depression	2.5 (3.4)	3.6 (3.6)	1.4 (2.4)	2.4 (2.8)	3.0 (3.7)	4.2 (3.8)
Anxiety	2.2 (2.3)	2.6 (2.4)	2.6 (2.9)	2.5 (1.9)	2.0 (2.0)	2.6 (2.6)
Stress	4.9 (3.4)	5.8 (3.6)	3.9 (4.0)	4.7 (3.2)	5.3 (3.0)	6.4 (3.7)
Physical Activity (IPAQ)						
Vigorous activity (min/week)	157.1 (328.5)	108.4 (191.8)	151.8 (282.2)	82.5 (110.9)	159.7 (353.6)	121.8 (223.0)
Moderate activity (min/week)	262.2 (387.4)	123.2 (235.9)	227.8 (230.5)	70.0 (78.4)	279.4 (448.2)	150.6 (282.8)
Walking (min/week)	479.8 (817.0)	317.6 (368.2)	306.6 (283.9)	362.8 (440.3)	566.4 (974.5)	292.2 (330.6)
Sitting (min/week)	395.6 (213.1)	417.8 (187.2)	433.1 (186.2)	470.6 (167.8)	376.9 (228.7)	390.6 (193.4)

*PSEQ* Pain Self Efficacy Questionnaire, *RMDQ* Roland Morris disability questionnaire, *NPS* numerical pain scale; *PSFS* patient-specific function scale; *IS* Insomnia Severity Index, *SCI* Sleep Condition Indicator; *PSQI* Pittsburgh Sleep Quality Index, *CBT-i* cognitive behavioral therapy for insomnia

Values in parentheses are standard deviations

^a^Ranges from 0 to 60; higher scores indicate better self-efficacy

^b^Ranges from 0 to 24; lower scores indicate lower disability

^c^Ranges from 0 to 100; lower scores indicate lower levels of pain

^d^Ranges from 0 to 10; higher scores indicate better function

^e^Ranges from 0 to 28; lower scores indicate less severity of insomnia

^f^Ranges from 0 to 32; higher scores indicate better sleep

^g^Ranges from 0 to 21; lower scores indicate better sleep quality

The singleton recruitment via the general community between October 2017 and August 2018, and the reasons for eligible participants discontinuing (*n* = 35) are described in Fig. 1. Most potential participants found out about the study via the NSW Seniors Cards’ newsletter (*n* = 212), followed by social media (*n* = 23). There were no costs pertained in their recruitment via the general community. Individual twins (*n* = 5) from the TRA that only met the eligibility criteria themselves and not their twin, were included in the singleton cohort.

#### Outcome measure completion, follow-up rate, and data collection

In our online surveys (REDCap), responses to most clinical outcomes were mandatory, which should have resulted in no missing values for participants who submitted the questionnaires (Table 3). However, the SCI was only considered as a follow-up outcome partway through the study, and therefore, 15 out of 23 twins who submitted the follow-up questionnaire did not have data for the SCI (total of 24 missing out of 32, 75%). The International Physical Activity Questionnaire Short Form [45] and PSQI were not mandatory, so some submitted questionnaire have missing responses to their outcomes. Some participants had partially completed but did not submit their questionnaires which resulted in a lower percentage of missing values compared to the percentage of people who were lost-to-follow-up or withdrew (Fig. 1). Overall, the total percentage of missing values across baseline, post-intervention, and follow-up were 13% for twins and 13% for singletons, which met our feasibility criteria (≤ 20%).

**Table 3:** Data collection and outcome completion rates

	Twins (baseline)	Twins (post-intervention)	Twins (follow-up)	Singletons (baseline)	Singletons (post-intervention)	Singletons
**Lost-to-follow-up/withdrew (%,** ***n*)**	0.00% (0)	18.75% (6)	28.13% (9)	0.00% (0)	21.21% (14)	21.21% (14)
PSEQ (%)	0.00%	9.38%	15,63%	0.00%	16.67%	18.18%
PSFS	0.00%	9.38%	15,63%	0.00%	16.67%	18.18%
NRS	0.00%	9.38%	15,63%	0.00%	27.27%	18.18%
RMPQ	0.00%	18.75%	28.13%	0.00%	21.21%	21.21%
IPAQ-SF	0.00%	12.50%	18.75%	15.15%	18.18%	22.73%
DASS	0.00%	12.50%	28.13%	0.00%	16.67%	21.21%
ISI	0.00%	12.50%	15.63%	0.00%	16.67%	19.70%
SCI	0.00%	12.50%	75.00%^a^	0.00%	16.67%	21.21%
PSQI	0.31%	18.75%	18.75%	1.59%	21.21%	21.21%
Total average	**0.03%**	**12.85%**	**25.69%**	**0.35%**	**19.02%**	**20.20%**
	**Twins**			**Singletons**		
Overall average	**12.86%**			**13.19%**		

PSEQ, PSFS, NRS, RMPQ, ISI, and SCI were mandatory for participants to submit their questionnaire responses, while the IPAQ-SF and PSQI were not

^a^The SCI was only considered as a follow-up outcome partway through the study, and therefore, 15 out of 23 twins who completed the follow-up questionnaire did not have data for the SCI (total 24 missing out of 32 = 75%)

Follow-up rates for the twins for the post-intervention and follow-up surveys were 81% and 72% respectively and for singletons 82% and 78% respectively and therefore did not meet our feasibility criteria (≥ 85%). This result was mainly due to lost-to-follow-up, although four participants withdrew from the study (Fig. 1). For the digital CBT-i groups, one twin withdrew due to “technical difficulties,” two singletons withdrew due to “no change or improvement in sleep” (*n* = 1), and “going overseas” (*n* = 1). All those who withdrew from the digital CBT-i group only completed the first session. For the control group, one singleton withdrew stating that their “situation had changed” and did not wish to discuss further. All participants who withdrew did not submit their responses for their post-intervention and follow-up questionnaires. There were no differences in the follow-up rates between the digital CBT-i and control groups at post-intervention or follow-up for twins (*p* = 0.31 and *p* = 0.35) and singletons (*p* = 0.31 and *p* = 0.92).

There were no major difficulties with using the REDCap software for data collection. Participants had no issues assessing the survey link via email. Five participants had initial difficulties answering certain questions in the right format which prevented the completion of the questionnaire, and this was rectified with the researchers. Three participants had trouble answering questions which used a slider scale on mobile devices, but this was resolved by using a computer instead. Phone interviews were conducted for 20 of the 32 twin participants (63%) at the end of the study, and all found the online questionnaires easy to understand, relevant, and acceptable in length of time to complete.

#### Contamination of intervention

The online responses for twins to assess contamination at follow-up are detailed in Table 4. From the questionnaire responses, four participants reported talking to their twin about the intervention they received (13%), two reported being aware of the intervention their twin received (6%), but none reported changing their behavior as a consequence of knowing their twin’s intervention. Therefore, the feasibility criterion of ≤ 15% being aware of the intervention their co-twin was receiving, was achieved.

**Table 4:** Contamination of intervention, adherence, and intervention credibility for the digital CBT-i and control groups

	Twins (digital CBT-i) (*n* = 16)	Twins (control) (*n* = 16)	Mean difference (95%CI)	Singletons (digital CBT-i) (*n* = 33)	Singletons (control) (*n* = 33)	Mean difference (95% CI)
**Contamination of intervention questions**						
Have you talked to your twin about the intervention you have received? *n*, Yes (%)	1 (6%)	3 (19%)				
Please indicate how confident you are that your twin did NOT know about the intervention you were receiving on a scale of 0 to 100, where 0 means “not at all” and 100 means “very confident”. Mean (SD)	86.7 (26.0)	85.5 (29.4)				
Were you aware of the intervention your twin was receiving? *n*, Yes (%)	1 (6%)	1 (6%)				
Did you change your behavior/attitude as a consequence of knowing about your twin intervention? *n*, Yes (%)	0 (0.00%)	0 (0.00%)				
**Adherence to digital CBT-i**, *n* (%)						
0 sessions	2 (13%)			2 (6%)		
1 sessions	1 (6%)			9 (27%)		
2 sessions	2 (13%)			2 (6%)		
3 sessions	1 (6%)			2 (6%)		
4 sessions	0 (0%)			0 (0%)		
5 sessions	1 (6%)			3 (9%)		
6 sessions	9 (56%)			15 (46%)		
4–6 sessions	10 (63%)			18 (55%)		
**Intervention credibility scale questions,** mean (SD)						
How confident do you feel that this intervention can help you cope with your sleep problems?^a^	3.75 (1.06)	2.80 (1.14)	0.95 (− 0.03–1.93)	3.17 (1.27)	2.18 (1.19)	0.99 (0.30–1.67)
How confident do you feel that this intervention will help you manage your sleep problems?^a^	3.92 (0.90)	2.42 (1.31)	1.50 (0.55–2.45)	3.29 (1.27)	2.04 (1.26)	1.26 (0.55–1.96)
How confident would you be in recommending this intervention to a friend who suffered from similar complaints?^a^	3.50 (1.00)	1.75 (1.48)	1.75 (0.68–2.82)	2.91 (1.56)	2.14 (1.30)	0.77 (0.03–1.58)
How logical does this intervention seem to you?^a^	4.18 (0.98)	3.83 (1.75)	0.35 (− 0.90–1.60)	3.96 (1.52)	2.39 (1.55)	1.57 (0.71–2.42)
Total score (0–24)	15.31 (3.29)	10.69 (3.43)	4.61 (1.76–7.46)	13.38 (4.93)	8.75 (4.71)	4.62 (1.94–7.31)

*CBT-i* cognitive behavioral therapy for insomnia

^a^Scores range from 0 (“not at all confident”) to 6 (“absolutely confident”)

In phone interviews with twins, two pairs (13%) reported discussing the study with each other and one pair had clearly shared what each of them received. Another pair reported noticing that her twin had different sleep habits to them. Two pairs reported living together, six pairs reported living in the same suburb and nine pairs reported communicating daily with each other.

#### Acceptability, credibility, and participants’ experience of the intervention

Ten out of 16 twin participants (63%) in the intervention group completed at least 4 of the 6 sessions of digital CBT-i, and for singletons, this was 18 out of 33 (55%) (Table 4). This did not meet the adherence feasibility criteria (≥75%).

At 1-week post-randomization, Total Intervention Credibility Scale Scores (0–24) were below 12 for control groups in the twin (mean = 10.69, SD = 3.43) and singleton cohorts (8.75, SD = 4.71) (Table 4). This suggests that participants did not find the control group credible. Total Intervention Credibility Scale Scores were higher in the digital CBT-i group compared to the control group, for both the twin (mean difference = 4.61, 95% CI [1.76–7.46]) and singleton cohorts (4.62, [1.94–7.31]). Both totals were above 12 and suggest that the digital CBT-i was credible. There were no significant differences in the responses to each of the four questionnaire items or the total score, between twins and singletons.

Answers to the blinding question asked at post-intervention also suggested that participants did not find the control group credible. If participant blinding has been maintained and if both groups were equally credible, then only 50% of participants should be able to guess their allocation. However, for the twin cohort, 82% of participants in the intervention group and 64% for the control guessed their group correctly, and for singletons, this was 77% and 85% respectively. These results were consistent with the overall impression from participant comments regarding their intervention at 1-week post-randomization and at post intervention (Table 5).

**Table 5:** Participant comments regarding their experiences and opinion of their intervention

Digital CBT-i (intervention)	Digital educational program (control)
**One-week post-randomization comments**	
“I know most things the program has discussed so far. At the moment the most helpful thing has been the sleep diary to know exactly how much I sleep.” “Access to the community adds value because you know you are not alone.” “The intervention has laid out sleep goals, however, yet to address back pain issues.” “The Sleepio program is confusing. It has only asked me to fill in a sleep diary, which is logical, but after a week of doing this there has been no follow-up.” “It’s early days but having some professional advice gives me some positivity towards assisting me find answers to why how & helping find the answers! I already feel as if there is a goal set in place to make this happen” “Found it very helpful this far” “At the moment I am still just creating a sleep diary so I am not sure what the ‘changes’ to my sleep behaviour I’m going to see.”	“As I have generally done lots of reading about sleep and how to get it I don’t think a newsletter intervention will help me improve my sleep as I have read the info in the first three newsletters in other formats.” “I only received one newsletter and it had a couple of interesting facts/information but nothing I felt that would help me improve my sleep. I actually can’t even remember what that information is now so I guess I didn’t really absorb it.” “Doesn’t seem like much of an intervention. Only some information (most of which general knowledge).” “Intervention seems to be based on information on activities that I so far practice - regular bed times, no TV, electronic devices on in bedroom etc. So unsure of objectives of this style of intervention” “I was skeptical at the start but when reading the facts they have changed my sleep habits a little” “The information is good and reiterates things I have heard before about sleep but unless I’m missing something that’s all it is. It doesn’t ask me to put any particular strategies in place so unless I’m proactive about it and choose to make some changes myself it won’t help.” “At this stage reading general information about sleep and some common sense suggestions isn’t necessarily improving my sleep. It’s making me more aware of sleep however not resolving any issues” “The facts were interesting. The tiredness during the day information particularly related to me.” “I was expecting more back specific related problem solving. The information you have put in the emails I already know about and it doesn’t fix or is related to my back sleeping problems” “Room temperature- as per newsletter 1- good tip. I feel much better in the morning.” “Unsure how reading about sleep issues helps me.” “To date, the suggestions and information received is same or similar to that I’ve already had” “I don’t feel that this is really going to work for me” “I am not accessing any intervention. I’m only being given general information about sleep.” “I’m not sure I’m getting all the information. I have opened the emails and found articles to read. I have read all of them but have not changed anything as I already had this information and was incorporating it into my routine. Is there something else I’m supposed to do?” “The information provided is interesting and some of it I’ve not heard before but thus far I don’t find it helpful in dealing with my waking up with pain.”
**Post intervention comments** (after being asked if they think they were in the intervention or control)	
“My lower back pain again in my opinion is directly linked to my sleeping issues of staying asleep.” “I found the sleep restriction hard and had short naps most days but it didn’t stop me sleeping at night and when I didn’t nap I had longer periods of time when I slept with being restless.” “I felt extremely fatigued undertaking the Sleepio course - more so than usual” “Could not get the video to open so could not complete full study” “I think I am concentrating a bit much on the sleep problem because I have to record every day. I usually try not to think about it so that it doesn’t become a problem.” “Was skeptical regarding Sleepio but although couldn’t assist with discomfort in bed gave some useful strategies.” “I feel the sleepio program has helped me as my back pain at night has decreased - the interventions to help with sleep help the back pain. I am pleased to have had the opportunity to go on the sleepio program and its outcome has been good for me. I will say that in the early weeks it was only the commitment to being a participant in a scientific trial that kept me going.” “I am settling down to sleep much better now that I follow the relax procedure and focus on something pleasurable (walking through a garden).” “My sleep has definitely improved over the 6 week period” “I believe it has helped my understanding more of sleep patterns. I no longer stress at not getting enough sleep that night as I night catch up the following evening plus I enjoyed the interaction with the Professor [avatar]” “The last week I have felt pain due I think to inflammation. This has had an impact on my sleep however overall I have experienced better sleep since being on the program” “In my view the advice on the room set up, exercise and reduced intake of caffeine before bedtime assisted greatly in improving my sleep quality. I also try to sit less at the Office and is using as standing desk. The standing desk definitely has an impact on back pain” “I will keep trying the techniques from the Prof [avatar] and see how they go”	“I actually found the newsletters very informative and I have put some into action. Like keeping more regular sleep patterns.” “Although the information in the newsletter was interesting, with no requirement to take action it doesn’t really change anything. Much of the information I had heard at some point or other. I am aware of the effect that technology before bed has on sleep, and how exercise can aid both sleep and back pain, it’s the following through on those things consistently that I struggle with. I feel there had been a commitment required to implement changes in relation to some of those factors then I would have seen beneficial results.” “Being control was not helpful to me, hope it was for the study.” “The newsletter seemed like common sense - reading irrelevant facts and figures about sleep was not going to help the problem” “I felt the tips on sleep were helpful, like going to bed at the same time, not being tempted to sleep during the day and the tips helped me to try and be more positive” “It was fairly obvious I was in the control group as it was just random facts about sleep. Nothing that could help me and there was no request for to actually do anything to change my behaviour.”
**At follow-up questionnaire/interviews**	
The participant reported liking the interactiveness of the program, and mindfulness strategies, and the extra resources and forums Sleepio has.	

*CBT-i* cognitive behavioral therapy for insomnia

There were six occurrences (6%) of technical difficulties where participants had trouble assessing the intervention. Three of these were difficulties in locating the link to online sessions, the other three with issues with access through mobile devices and video playback (i.e., “[I] could not get the video to open”). While digital CBT-i users had an option to contact the digital intervention’s own technical support, these participants reported their difficulties to the researchers and the researchers troubleshooted all these cases. The remainder of the participants in the intervention group had reported no difficulties accessing the digital intervention.

Overall, participants had a positive experience with the digital CBT-i intervention. Out of 21 comments, eleven were positive, five were neutral, and six were negative (Table 5). Positive experiences mainly included the comments regarding improvements in sleep (*n* = 5), improvements in pain (*n* = 1), and the interactiveness of the program (*n* = 2). In comparison, of the control group feedback, five were positive, three were neutral, and fourteen were negative. Adverse events were not explicitly evaluated in the present study due to the relatively safe nature of the CBT-i intervention; however, one participant reported more fatigue than usual.

## Discussion

### Feasibility summary

A trial exploring the efficacy of a digital CBT-i in people with comorbid symptoms of insomnia and LBP over 6 weeks with 3 months follow-up found that the intervention was accessible but not fully feasible in its current state for twins or singletons. For twins, feasibility goals were met for contamination of intervention and data collection, but not for recruitment, follow-up, and adherence. For singletons, the criteria for follow-up rate and adherence rate were not met. For this trial to be feasible for twins or singletons, several trial design strategies may need to be implemented.

### Recruitment rate

The recruitment rate for twins (16 pairs over 3 years) may have been higher if recruitment strategies were fully focused on the present study. The recruitment of twins from TRA initially advertised both the AUTBACK [36] study and the present study (SleepBack) and gave twins the choice to participate in either study. The low recruitment rate (2.2%) may have been attributed to participants having more interest in the AUTBACK study as it did not have insomnia as an inclusion criterion for both twins. Financial costs were a limiting factor for recruitment, as there were costs per twin pair (AUD 9) for invitational emails, phone calls, follow-up, and administration. Therefore, in our protocol, we only had the TRA directly approach twins which reported having LBP for > 6 weeks in a 2014 TRA questionnaire and this targeted approach may have many twins who did not currently have LBP. Potentially as technology improves the costs to invite participants will decrease and enable faster recruitment for the same budget.

### Adherence, control group credibility, and follow-up rate

Adherence rates to the digital CBT-i sessions (55–63%) were lower than previously reported trials [21, 22] of Sleepio for people with symptoms of insomnia only, where 58–85% completed ≥ 4 out of 6 sessions. Adherence rates were also lower than a face to face CBT program which included both insomnia and pain components, for adolescents with comorbid migraine and insomnia [46].

While participants were not asked about reasons for non-adherence, we hypothesize several potential reasons for this difference in adherence rates in both groups. Our participants were people with comorbid symptoms of insomnia and LBP, and this comorbidity may made adherence more difficult due to widespread effects of pain on emotional, cognitive, and physical function [47]. More importantly, some participants were not primarily seeking care for insomnia. While our interventions for our experimental (Sleepio) and control group (education) were designed to target insomnia only, some participants expected pain to be directly addressed (“The intervention has laid out sleep goals, however, yet to address back pain issues.”). Therefore, to improve adherence for the experimental group, the digital CBT-i may need to be tailored to provide pain advice and education so that both insomnia and LBP are targeted.

In the control group, the credibility scores and comments such as “Doesn’t seem like much of an intervention. Only some information (most of which general knowledge)” indicated poor acceptability of the treatment. A more credible control may have been needed, as one participant said “It was fairly obvious I was in the control group as it was just random facts about sleep. Nothing that could help me and there was no request for to actually do anything to change my behavior.” Instead of an educational email newsletter, a digital application which delivers general information but also requests participants to record a sleep diary might be a more credible control.

In the present study, poor adherence to the digital CBT-i and poor credibility of the control group may have also potentially reduced the follow-up rate. Addressing these with the above suggestions may partially rectify this. Other ways to improve follow-up rates may include (1) altering the reminder system and (2) building better rapport with participants. Instead of email reminders with the questionnaire link on the day, text messages at 7 days, and phone calls at 14 days, it may be more effective to have both the email and text reminder messages with the questionnaire link on the day [48]. Improving the closeness of the survey completion time to the measurement period will also ensure better accuracy of the outcomes. Periodical text messages (e.g., fortnightly) to check up on participants on their progress with their intervention, might be used to improve rapport and follow-up rate

### Strengths and limitations

The major strength of the present study is the randomized controlled trial design which included concealed allocation, blinded outcome assessment, blinded analysis, intention-to-treat analysis, and the prior publication of the protocol. Online electronic surveys have ensured more potentially cost-effective and accurate measurements of adherence, credibility, and clinical outcomes compared to handwritten surveys. We have included cohorts of twin and singletons for relevant comparison of feasibility. The present study has been reported following the CONSORT statement [34] and TIDieR checklist [35].

There were several limitations in the present study. Firstly, most participants were contacted via telephone (twins) or online (twins and singletons), which may represent a cohort which was more interested in addressing insomnia and have greater access and competencies in using digital platforms, compared to the wider population. Secondly, while the diagnosis of insomnia assumes the absence of other sleep disorders, we did not rule out other sleep disorders via polysomnography measurements due to costs [13]. However, digital CBT for insomnia may work for insomnia symptoms even when they co-present with other sleep disorders [49]. Thirdly, the follow-up rate may have been influenced by the amount of attention participants received, as the control group received little attention (sleep education emails) compared to the interactiveness of Sleepio and its online community. Fourthly, it is unknown what proportion of the control participants read the educational emails as this was not monitored. This may have explained the lower credibility scores for the control intervention. Finally, the digital CBT-i (Sleepio) intervention consisted of multiple components, and therefore, it not possible to determine which component (e.g., sleep information, sleep restriction, sleep hygiene, mindfulness) was most acceptable and credible to participants. In light of the feedback from participants and study limitations, further research should explore whether there is a benefit in tailoring digital CBT-i to pain conditions and whether certain individual components of digital CBT-I are more beneficial or even detrimental compared to others.

## Conclusion

The present study provides evidence that digital CBT-i sleep intervention for people with comorbid symptoms of insomnia and LBP is accessible, and overall participants had a good impression of the intervention. Despite the successful online data collection, the study in its current form has limited feasibility unless strategies to improve adherence, follow-up, control group credibility, and twin recruitment rates are implemented.

## Supplementary Information

**Additional file 1.** Summary of treatment characteristics.

**Additional file 2.** CONSORT Checklist.

**Additional file 3.** TIDieR Checklist.

## Data Availability

The datasets generated and/or analyzed during the current study are not publicly available due data linkages to Twins Research Australia but are available from the corresponding author and Twins Research Australia on reasonable request.

## Abbreviations

CBT-I: Cognitive behavioral therapy for insomnia
CI: Confidence intervals
LBP: Low back pain
PSQI: Pittsburgh Sleep Quality Index
REDCap: Research Electronic Data Capture
SCI: Sleep Condition Indicator
TRA: Twins Research Australia
