# Supplementary material for: Efficacy of a digital cognitive behavioral therapy for insomnia in people with low back pain: a feasibility randomized co-twin and singleton-controlled trial
Source: Pilot Feasibility Stud. 2022 Jun 14;8:125. doi: 10.1186/s40814-022-01087-z (PMC9195289; doi:10.1186/s40814-022-01087-z)
Supplement: Supplementary file 1 — Additional file 1. Summary of treatment characteristics. [file 40814_2022_1087_MOESM1_ESM.docx]

**Appendix 1.** Summary of treatment characteristics

|  | **Digital Cognitive Behavioral Therapy (Sleepio)** | **Educational Control** |
| --- | --- | --- |
| **Treatment content** | Sleep information/education, sleep hygiene, relaxation, stimulus control, sleep restriction, cognitive techniques (restructuring, paradox, mindfulness, imagery, putting day to rest, thought stopping) | Sleep information (with sleep hygiene tips) delivered by email not in a personally tailored manner |
| **Duration** | Minimum of 6 weeks (1 session/week) | 6 weeks (one email/week) |
| **Additional treatment characteristics** | Appointment system, interactive sessions, dynamic feedback against personal goals, review of the progress at the beginning of each session, automatic calculation of sleep data over time, personal case file, quiz at the end of the sessions, 24/7 access | N/A |
| **Support / motivational system** | Reinforce contingent on progress, access to online material on sleep educational topics, social community of users (moderated by experts), support/reminders/prompts by email and mobile SMS, “graduation ceremony” on course completion | Email support only |

N/A: not applicable.
